# Usability Evaluations of a Wearable Inertial Sensing System and Quality of Movement Metrics for Stroke Survivors by Care Professionals

**DOI:** 10.3389/fbioe.2017.00020

**Published:** 2017-04-03

**Authors:** Bart Klaassen, Bert-Jan F. van Beijnum, Jeremia P. Held, Jasper Reenalda, Fokke B. van Meulen, Peter H. Veltink, Hermie J. Hermens

**Affiliations:** ^1^Biomedical Signals and Systems, MIRA – Institute for Biomedical Technology and Technical Medicine, University of Twente, Enschede, Netherlands; ^2^Centre for Telematics and Information Technology, University of Twente, Enschede, Netherlands; ^3^Division of Vascular Neurology and Neurorehabilitation, Department of Neurology, University Hospital of Zurich, Zurich, Switzerland; ^4^Cereneo, Center for Neurology and Rehabilitation, Vitznau, Switzerland; ^5^Roessingh Research and Development, Roessingh Rehabilitation Hospital, Enschede, Netherlands; ^6^Biomechanical Engineering, MIRA – Institute for Biomedical Technology and Technical Medicine, University of Twente, Enschede, Netherlands

**Keywords:** stroke, rehabilitation, inertial sensing, daily life, data processing, technology assessment, evaluation, usability

## Abstract

**Background:**

Inertial motion capture systems are used in many applications such as measuring the movement quality in stroke survivors. The absence of clinical effectiveness and usability evidence in these assistive technologies into rehabilitation has delayed the transition of research into clinical practice. Recently, a new inertial motion capture system was developed in a project, called INTERACTION, to objectively measure the quality of movement (QoM) in stroke survivors during daily-life activity. With INTERACTION, we are to be able to investigate into what happens with patients after discharge from the hospital. Resulting QoM metrics, where a metric is defined as a measure of some property, are subsequently presented to care professionals. Metrics include for example: reaching distance, walking speed, and hand distribution plots. The latter shows a density plot of the hand position in the transversal plane. The objective of this study is to investigate the opinions of care professionals in using these metrics obtained from INTERACTION and its usability.

**Methods:**

By means of a semi-structured interview, guided by a presentation, presenting two patient reports. Each report includes several QoM metric (like reaching distance, hand position density plots, shoulder abduction) results obtained during daily-life measurements and in clinic and were evaluated by care professionals not related to the project. The results were compared with care professionals involved within the INTERACTION project. Furthermore, two questionnaires (5-point Likert and open questionnaire) were handed over to rate the usability of the metrics and to investigate if they would like such a system in their clinic.

**Results:**

Eleven interviews were conducted, where each interview included either two or three care professionals as a group, in Switzerland and The Netherlands. Evaluation of the case reports (CRs) by participants and INTERACTION members showed a high correlation for both lower and upper extremity metrics. Participants were most in favor of hand distribution plots during daily-life activities. All participants mentioned that visualizing QoM of stroke survivors over time during daily-life activities has more possibilities compared to current clinical assessments. They also mentioned that these metrics could be important for self-evaluation of stroke survivors.

**Discussion:**

The results showed that most participants were able to understand the metrics presented in the CRs. For a few metrics, it remained difficult to assess the underlying cause of the QoM. Hence, a combination of metrics is needed to get a better insight of the patient. Furthermore, it remains important to report the state (e.g., how the patient feels), its surroundings (outside, inside the house, on a slippery surface), and detail of specific activities (does the patient grasps a piece of paper or a heavy cooking pan but also dual tasks). Altogether, it remains a questions how to determine what the patient is doing and where the patient is doing his or her activities.

## Introduction

Inertial motion capture systems are used in many applications such as measuring the quality of movement (QoM) in patients with neurological conditions, seen as assistive technologies in healthcare, and capturing body movements, for e.g., film animation and games (Bauer et al., 2012; Kitagawa and Windsor, 2012; Altilio et al., 2015; Klaassen et al., 2015a) Recently, a new ambulant full-body inertial motion capture system was developed in a project, called INTERACTION, to objectively measure the QoM in stroke survivors during daily-life activities (Klaassen et al., 2015a; Lorussi et al., 2016; van Meulen et al., 2016). Daily-life monitoring of the QoM during functional activities of stroke survivors in their home environment is important, as it is essential for optimal guidance of rehabilitation therapy (Kollen et al., 2006).

Although (full body) motion capture systems are used in some specialized clinics for a better understanding of patients, such as Qualisys mocap systems (Qualisys, 2008), the absence of clinical effectiveness and usability evidence in assistive technologies, e.g., motion capture and telemonitoring systems, into rehabilitation has delayed the transition of research into general clinical practice (Burridge and Hughes, 2010; Bergmann and McGregor, 2011). The potential for these technologies in clinical practice is determined by a complex interaction between clinical and cost-effectiveness; commercial availability of devices; funding and user acceptance including patients, their careers, healthcare professionals, funders, and manufacturers (Burridge and Hughes, 2010; Bergmann and McGregor, 2011). Furthermore, key barriers that were found to translate assistive technologies into clinical practice are lack of knowledge, education, awareness, and access. Improvements in assistive technology design, clinical evaluation, knowledge, awareness, and in provision of services will contribute to a better and cost-effective stroke rehabilitation (Hughes et al., 2014). Therefore, it is useful to perform a field study to investigate if care professionals from other rehabilitation centers, not involved in the project, are able to use the outcome measures from an inertial motion capture system, like the INTERACTION system, in their clinical practice.

The INTERACTION system enables telemonitoring capabilities by constructing an architecture capable of capturing movement data, storing data remotely, processing the data, and finally visualizing patient data in a report form. Resulting outcome metrics of the system enables new insights into the differences between in-clinic and outpatient measurements of stroke survivors over longer periods of time (van Meulen et al., 2016). The final system is composed of a full-body sensor suit with 14 inertial sensors specifically designed for stroke survivors to be measured at home during daily-life activities (Klaassen et al., 2015a). It consists of a shirt, trousers, and a pair of shoes and is wireless connected to a tablet with the functionality to upload the data to a remote server for data processing. Although the INTERACTION system consists of different types of sensing modules, only the inertial sensors are used to compute the resulting metrics included in this study, as presented in van Meulen et al. (2016). A software package was developed to analyze the data, which includes an activity monitor, movement visualizer, and several metrics for the QoM. The package generates case reports (CRs) for each patient measurement as described in van Meulen et al. (2016).

This system was used for an extended period of time to monitor stroke survivors at home to gain new insights into the performance of these patients during daily-life activities. The technological concept of INTERACTION system was fully implemented in daily-life, where some issues related to, e.g., magnetic distortions and wireless capture range had to be overcome in order to achieve the highest possible data quality. Both were to be expected during daily-life usage with wireless inertial-based motion capture system relying on magnetometers for the positional estimation of body segments (Klaassen et al., 2015a; van Meulen et al., 2016).

In this paper, we focus on the resulting QoM metrics of the system and how care professionals might integrate it in current clinical practice. The objective of this study therefore is to investigate the opinions of care professionals in using metrics obtained from INTERACTION system during clinical and daily-life measurements and their usability. In particular, the focus of this study is on the interpretation of the QoM metrics for lower and upper extremity, compared to existing clinical methods and if a system like INTERACTION, including a full-body suit with many sensors, can be used in a variety of clinical practices. By means of a semi-structured interview, the evaluation of two CRs by care professionals involved in INTERACTION was compared with the evaluation of care professionals outside the project. Furthermore, two questionnaires were handed over to rate the metrics of extremity function in terms of their liking and an open questionnaire was given to investigate if they would adopt such a full-body motion capture system for their clinical practice.

## Materials and Methods

Within the INTERACTION project, patient’s CRs were constructed. Each report consists of results of a stroke survivor measured with the INTERACTION system in the form of metrics (Klaassen et al., 2015a; van Meulen et al., 2016). Within each set of INTERACTION measurements, the patient’s QoM was estimated at home, performing daily activities, and in clinic during frequently used clinical tasks: Timed-Up-and-Go (TuG) (Podsiadlo and Richardson, 1991) and the upper extremity part of the Fugl–Meyer Assessment (FMA-UE) (Gladstone et al., 2002). The measurements were part of a larger study from INTERACTION (Tognetti et al., 2014; Veltink et al., 2014; van Meulen et al., 2016). Care professionals involved in the INTERACTION project evaluated these CRs. During a semi-structured interview, two of these CRs, one for the lower extremities and one for the upper extremities, were presented to care professionals from outside the project. Furthermore, a ranking questionnaire and an open questionnaire were given to gain more insight in the use of inertial motion capture systems, such as the INTERACTION system, in clinic and the resulting metrics for therapy.

### Case Reports

**Figure A1:**
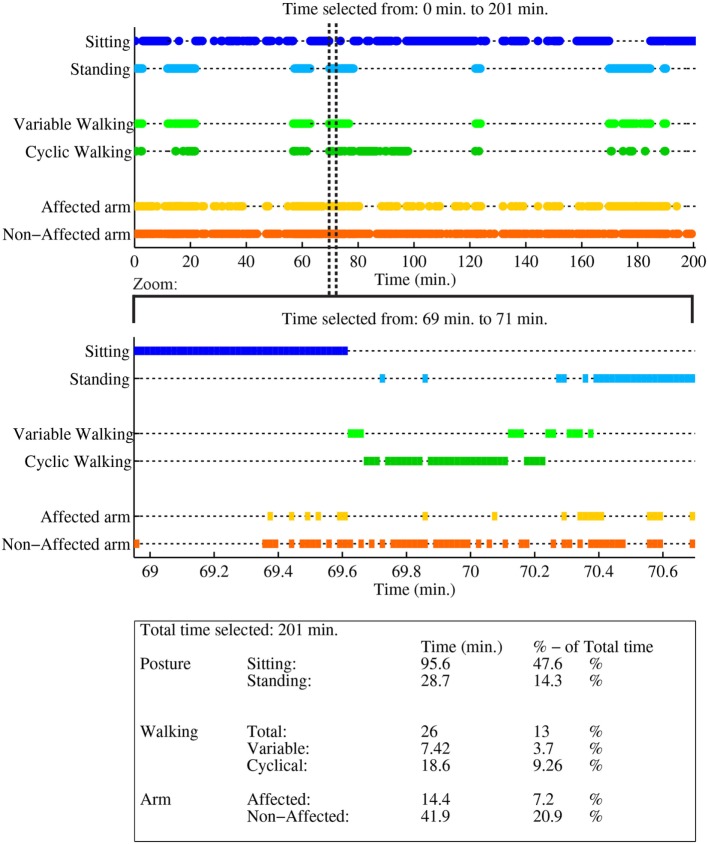
**Case report example page 1: activity monitor, as reported in van Meulen et al. (2016)**.

**Figure A2:**
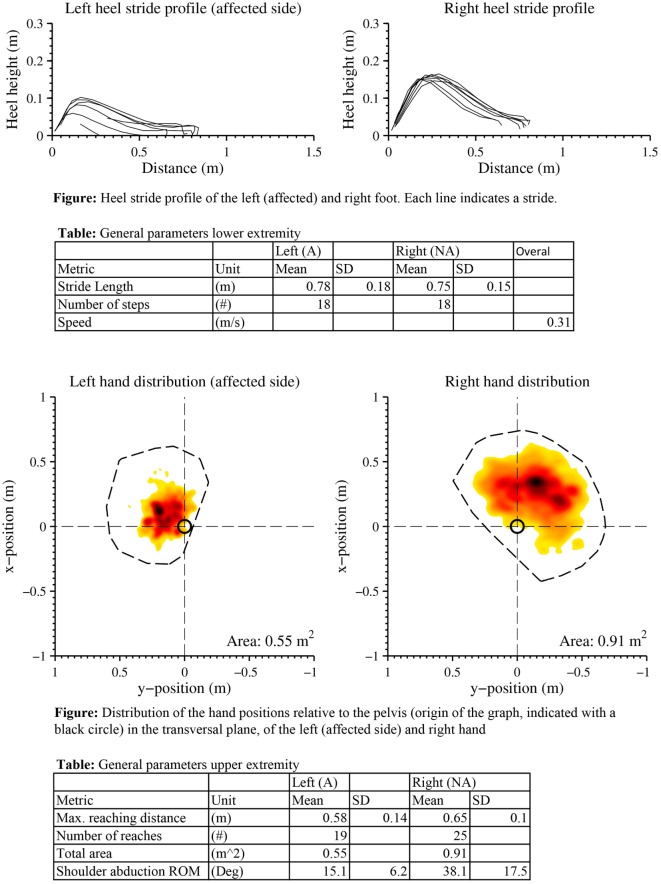
**Case report example page 2: a selection of metrics of extremity function from one measurement, as reported in van Meulen et al. (2016)**.

Two CRs were generated, where measurement sessions were selected within the rehabilitation program of two patients to construct the CR. One for the lower extremity (CR1) and one for the upper extremities (CR2). The study protocol for these patient measurements is a subset of a larger protocol that was approved by the local cantonal medical and ethical committee (registered in http://ClinicalTrials.gov identifier: NCT02118363). Each report includes the results, in the form of metrics, of a stroke survivor measured with the INTERACTION system in clinic during several days, and at home over a time period of 3 months. In addition, traditional clinical assessment scores of the patient were obtained from the clinics. The upper and lower extremity metrics were presented in van Meulen et al., 2016. In short, the following metrics were included for the lower extremities: heel stride profiles, which shows the heel height and stride length plotted for each stride, and walking speed. For the upper extremity metrics the following metrics were selected: reaching distance (hand-sternum distance), reaching area (area in which the patient moves their hand in transversal plane), and range of motion of the shoulder and hand distribution plots. Hand distribution plots are a new type of metric designed for INTERACTION that shows spatial and temporal information of the hand position relative to the pelvis in the transversal plane. The color shown in these distribution plots are mapped to the duration, the hand was positioned in a specific location. No activity monitor results, as described in van Meulen et al., 2016, were presented to reduce the amount of information to the participants. An example of a CR is shown in Appendix 1, Figures A1 and A2.

### Evaluation Protocol

**Figure 1:**
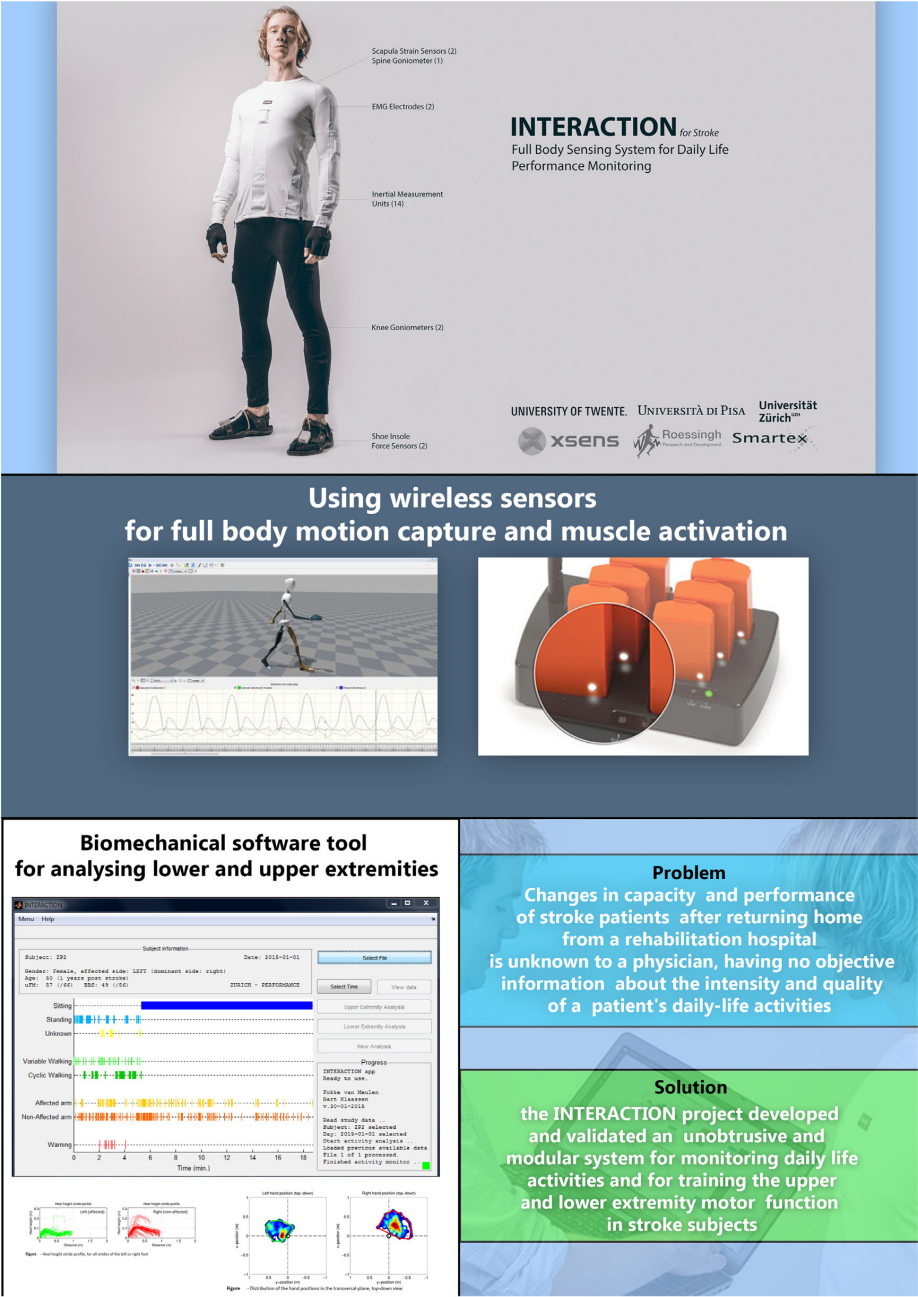
**Flyer of the INTERACTION project showing the sensor suit, sensors, software (with activity monitor and case report generator), and the main objectives of the INTERACTION project and solutions**.

**Figure 2:**
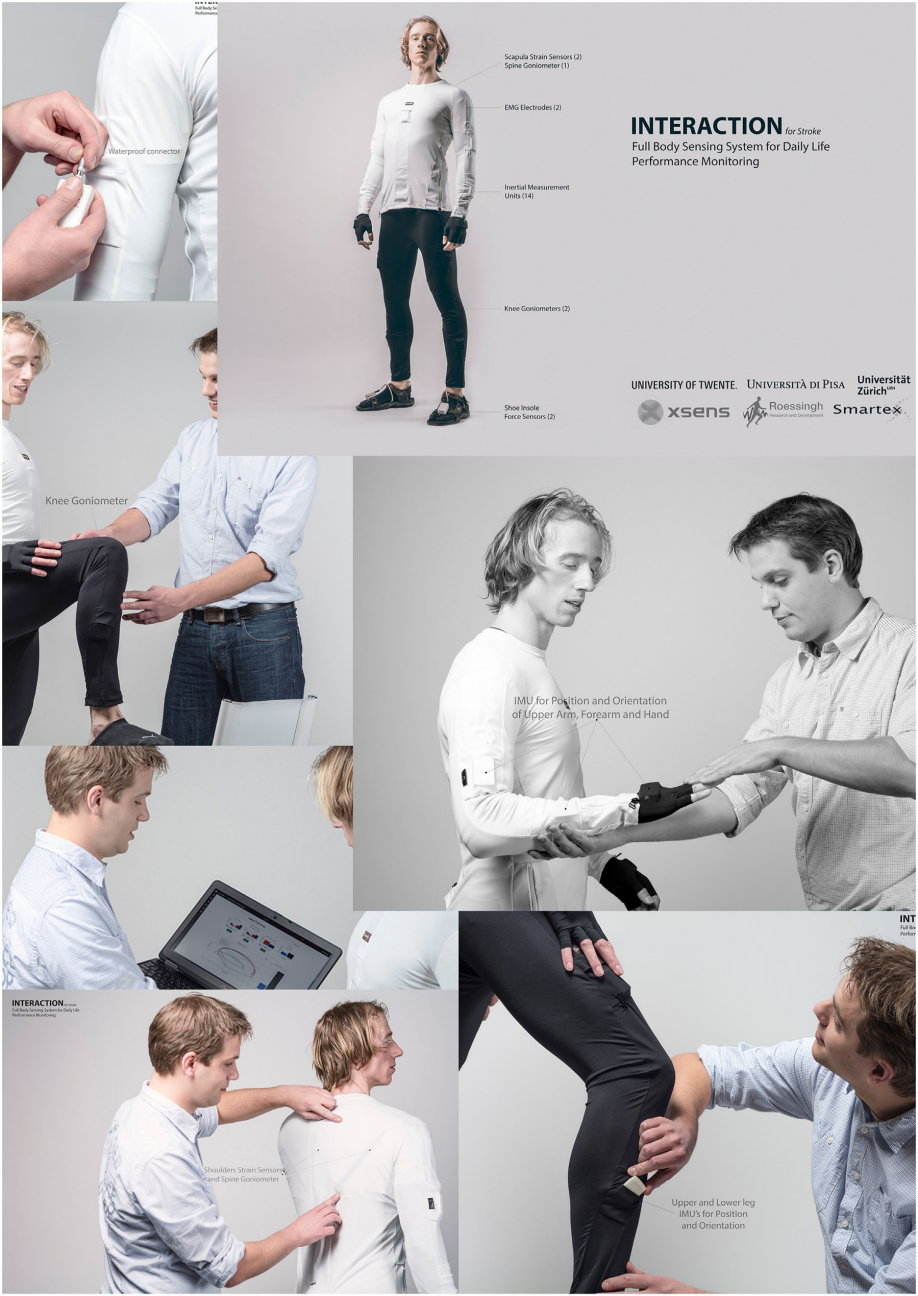
**Flyer of the INTERACTION project showing several pictures of the sensor suit and the web-portal for displaying quality of movement metrics**.

Each interview was conducted by the same person, following the four steps mentioned below. The interviews were captured on a digital field recorder for later analysis. First, information about the INTERACTION project, including a flyer about the system (with information), and a description of this study was handed over as shown in Figures 1 and 2. Second, an inform consent form was presented and explained to the care professionals. After informed consent was given, the evaluation started and the digital field recorder was turned on. Third, a semi-structured interview was held, guided by a presentation on a laptop, and aimed to last 1 h. Finally, a 5-point Likert questionnaire (Allen and Seaman, 2007) and an open questionnaire were filled in.

### Semi-Structured Interview and Presented Metrics

The semi-structured interview was supported by a PowerPoint presentation. The slides were organized as follows: first, the clinical assessment metrics were shown, then an INTERACTION metric from a measurement in clinic was shown, and finally the same metric captured during activities of daily living (ADL) at home was shown. This process was repeated for all the metrics mentioned in Section “Case Reports”. An example of each metric was shown and explained to the participants before the metric with patient data (in clinic or at home) were revealed. In detail, the origin of the data, the type of data, and the graphs axis were explained. Subsequently, three questions were asked for each metric:

- What do you see?
- What does it add for you compared to structured clinical measures?
- Is there any downside to this new metric?

### Likert Questionnaire and Open Questionnaire

After the semi-structured interview, a standard 5-point Likert questionnaire (Allen and Seaman, 2007) was handed over to rate the presented metrics with a score of 0 points (do not want to have) to 5 points (want to have). Finally, a non-standardized open questionnaire was presented about using the system and metrics in clinic, with the following questions:

- Do you want to have an inertial motion capture system, like INTERACTION, in your clinic?
- Do you have enough resources (e.g., time and money) in your clinic to utilize this system?
- Do you have any additions to what can be measured?

### Reference Analysis

For comparing the evaluations of care professionals (in and outside the project), a feature list was constructed, where a feature (F) is a conclusion given by the care professional on resulting metric in the patient-report (Table 1). A score per feature (as shown in Table 1), were assigned by researchers from INTERACTION. This score provides a weight to each feature based on that features complexity. In total, 10 features were defined, based on the patient CR, four (Fl_1–Fl_4) for the lower extremity CR, where Fl_1 is based on tradition clinic assessments and Fl_2 on measurement data captured by INTERACTION in clinic and Fl_3 and Fl_4 during daily life. For the upper extremity CR, six features (Fu_1–Fu_6) are included, as listed in Table 1, where Fu_1 is based on traditional assessments, Fu_2 and Fu_3 are captured in clinic and Fu_4–Fu_6 during daily life with the INTERACTION system. Each feature, except for Fl_1 and Fu_1, is obtained using the INTERACTION system. The evaluation of both CRs by care professionals during the interview was captured and points were given if a feature was mentioned. As a maximum, 50 points could be assigned.

**Table 1:** **Feature reference list**.

Case report (CR) 1: lower extremities		
Feature points details		
Fl_1^a^	5	Based on TuG assessment scores, see an improvement in function of gait
Based on in-clinic measurements (C)		
Fl_2	2	Observed difference in stride profile metric between left/right side over time for clinical task (TuG) between left/right side over time for clinical task (TuG)
	2	Indicated that difference disappeared over time for clinical task (TuG)
	1	Showing an improvement of foot clearance during clinical task (TuG)
Based on daily-life measurements (D)		
Fl_3	2	Observed a difference in heel height between left/right in ADL walking tasks
	3	Observed no change over time during ADL walking tasks
Fl_4	3	Noted improvement of walking speed in clinical task (TuG)
	2	Observed no change in walking speed during ADL walking task

**CR 2: upper extremities**		

Fu_1^a^	5	Based on the FMA-UE assessment scores, show no distinct improvement over time
Based on in-clinic measurements (C)		
Fu_2	2	Observed an increase in reaching distance of affected side between baseline and discharge, but then a decrease back to baseline for clinical task (FMA-UE)
	3	Observed an increase in total area/hand distribution plots of affected side between baseline and discharge, but then a decrease back to baseline UE for clinical task (FMA-UE)
Fu_3	5	Showing that abduction of shoulder increased between baseline and discharge, but then decreased back to baseline of affected and non-affected arm for clinical tasks (FMA-UE)
Based on daily-life measurements (D)		
Fu_4	3	Noted that shoulder abduction ROM was higher for the non-affected arm than for the affected arm during ADL
	2	Indicated no clear improvement of shoulder abduction over time during ADL
Fu_5	2	Show little change in max reaching distance over time during ADL
	3	Show little change in total area over time during ADL
Fu_6	5	Total reaching area was lower for the affected side than for the non-affected side during ADL

*Points (P) are assigned to each feature for the lower extremities (Fl_1, Fl_4) and upper extremity (Fu_1–Fu_6)*.

*^a^Obtained via clinical assessments scores, all other features are obtained with the INTERACTION system*.

*TuG, Timed-Up-and-Go assessment task (Podsiadlo and Richardson, 1991); FMA-UE, upper extremity part of the Fugl–Meyer assessment (Gladstone et al., 2002); ADL, activities of daily living*.

### Data Processing

The captured semi-structured interviews were transcoded in Atlas TI 2.0 (Muhr, 2004). The transcoded interviews were analyzed for the reference analysis. Means and SDs of the Likert questionnaire scores were calculated per INTERACTION metric.

### Participants

Care professionals were recruited from five different treatment centers: Roessingh Rehabilitation Center (Enschede, The Netherlands), Cereneo Clinic, Center for Neurology and Rehabilitation (Vitznau, Switzerland), University Hospital Zurich (Zurich, Switzerland), Physiotherapy Praxis Meilen (Herrliberg, Switzerland), and Fysio Holland (Enschede, The Netherlands). These centers were selected because of the expertise they provide in rehabilitation of stroke survivors. In this study, care professionals had no past involvement with the INTERACTION project, but needed to have experience with stroke survivors and are actively involved in rehabilitation. The decision was made to perform the semi-structured interview with care professionals in pairs, to encourage discussions which could lead to potentially more insight in the data. Written informed consent was given, but no ethical approval was needed as no medical intervention took place. In total, 11 interviews were conducted with 11 groups consisting of 23 participants. Seven interviews were held in Switzerland divided over three institutes. Six interviews consisted of two care professionals and one interview was conducted with three care professionals. Four interviews were held in The Netherlands divided over two institutes. Each interview group consisted of two care professionals. The interviews were held between July 2015 and October 2015. The interviews, on average, lasted 51 ± 11 min and were all successfully completed. All participants filled in the questionnaires at the end of the interview.

## Results

### Reference Analysis Results

The results of the reference analysis are listed in Table 2. The participants scored, on average, 47.3 ± 3.5 points. For the lower extremity case report (CR1), they were able to extract each feature. For the upper extremity case report (CR2), participants scored lower for descriptive statistics of reaching distance over time for both in clinic and at home.

**Table 2:** **Reference analysis results**.

	CR1^a^				CR2						
	
Location^b^	C	C	D	D	C	C	C	D	D	D	
	
Features	Fl_1	Fl_2	Fl_3	Fl_4	Fu_1	Fu_2	Fu_3	Fu_4	Fu_5	Fu_6	Total
**# interviews^c^**											
INT S1	5	5	5	5	5	5	5	5	5	5	50
INT S2	5	5	5	5	5	5	5	5	5	5	50
INT S3	5	5	3	5	5	5	5	2	5	5	45
INT S4	5	5	5	5	5	5	5	5	5	5	50
INT S5	5	5	5	5	5	2	5	5	2	5	44
INT S6	5	5	5	5	5	5	5	5	5	5	50
INT S7	5	5	5	5	5	5	5	5	5	5	50
INT NL1	5	5	5	5	5	5	5	5	5	5	50
INT NL2	5	5	5	5	5	2	5	2	5	2	41
INT NL3	5	5	5	5	5	1	5	5	0	5	41
INT NL4	5	5	5	5	5	5	5	5	5	5	50
Average	5	5	4.8	5	5	4.1	5	4.5	4.3	4.7	47.4
Std	0	0	0.6	0	0	1.6	0	1.2	1.7	0.9	3.8

*^a^Case report (CR) 1: lower extremity, 2: upper extremity*.

*^b^Based on in-clinic (C) or during daily-life (D) measurements*.

*^c^Interview (INT) held in Switzerland (SX) and The Netherlands (NLX)*.

### Interview Results

#### What Do You See?

Participants were asked to interpret the metrics shown for each CR. Of 11 groups, 7 (64%) were able to relate data shown in each metric with movement disorder or issues of the patient for the upper extremities, where all groups could do it for the lower extremities. The four groups who had difficulties relating the metric results to movement disorders for the upper extremities found it difficult to describe what the metric actually showed them in relation to functional movement of the patient. This applied in particular to the metrics: reaching distance and shoulder abduction range of motion. The participants explained that with these metric results, they did not know exactly how the patient managed to get to that result. Therefore, they could not describe the underlying cause of particular movements or give suggestions for future therapy. In total, six groups were able to give a detailed description of the hand distribution plots of the hand during daily-life activities, including compensation strategies, certain movements the patient performed, and insight in what these patients should train. For example, introduce a new therapy to enhance the patient’s ability to reach more sideways. The other five groups were able to understand the density plots, but only mentioned differences in density plot surface areas.

#### What Does It Add for You?

In total, 9 out of 11 groups mentioned that they prefer objective measures for the QoM as presented in the INTERACTION metrics compared to current clinical scores measured with a stopwatch or subjectively scored by a care professional. They expect that it will give them specific information about the patient’s movements and that the objective measures more accurate than current clinical scores. All groups mentioned that visualizing QoM by using these metrics, especially during daily-life activities, have more possibilities compared to clinical assessments. Especially, when compared over time, which makes it possible to intervene earlier before patients come back to the care professional with complains. Two groups mentioned that the INTERACTION system and resulting metrics could be a solution in the case you cannot physically see the patient in clinic. According to eight groups, they do not have to be an expert to understand the metrics, in particular the density plots of the hand. In these graphs, they see what patients are doing and what areas they have to work on. Two groups mentioned it would also help to show the progress of rehabilitation to insurance companies as proof that the patient is progressing, in order to reimburse their medical costs.

All groups mentioned in detail that feedback toward the patient is important and that these metrics, with particular focus on the density plots and reaching areas, are an important part of self-evaluation. One group also mentioned that patients often loose feeling of progress, particularly at home. Therefore, presenting those patients with weekly updated metrics about the QoM at home (most in favor: density plots) could help them regaining that feeling. These metrics captured in clinic and at home are of interest especially for reward-addicted patients according to two groups. Two groups stated that by just wearing such a system at home could be motivating to patients. Three groups mentioned that the shoulder range of movement is a compact and objective measure which can easily be compared over time. In total, 10 out of 11 groups agreed that the density plots are the most interesting metrics to summarize daily-life function at home and to visualize differences between the left and right arm. They mentioned that it would be a great metric to be used for clinical assessments, especially in rehabilitation clinics, but not in acute hospitals as these patients are not monitored for longer periods of time.

#### Are There Any Downsides to These New Metrics?

According to all groups, the density plots constructed based upon the clinical measurements were more difficult to read compared to density plots constructed from the daily activities at home, especially when trying to compare the left and right reaching area of the patient. In total, 9 out of 11 groups stated that the context of the home measurements is of importance in understanding the movement data. Distractions at home (such as tv, phone or other people), dual tasks the patients might perform or cognitive challenges all influence the movement performance of the patient. Furthermore, the environment, e.g., the surface of the ground, is of great influence to, for example, the movement speed or stride profile metrics. A clinical environment is controlled and safer than at home and therefore it remains difficult to compare clinic and daily-life situations. Three groups mentioned that the coaching or motivational aspect of a care professional being present during clinical assessments has a large influence on the capabilities of the patient during the measurement. Therefore, it might be challenging to compare these clinical results with a home situation. Five groups mentioned that it is not possible to assess how the patients manages to do the movement by only using the reaching distance or shoulder range of motion metric. Including information like: carrying an object, grasping, or muscle activation would help in understanding how patients are able to perform a certain movement. Concerning the lower extremity stride profiles, two groups stated that it is not possible to properly assess how the patient is making his foot movement (if he drags the foot or swings it outside) which affects the patient’s therapy. One group stated they would like to have the metric values for movements made by healthy subjects as a reference. One group mentioned they want to see the selected metrics only for a very specific activity.

### Likert Questionnaire Results

The metrics, as described earlier, were scored by each group of care professionals from 0 (not to have) to 5 (really want to have). The results are listed in Table 3. Hand distribution plots, constructed from ADL at home and for the FMA-UE, were the most favorite metric to have, with a mean of 4.2 ± 1.2 out of 5. Next is the reaching area descriptive statistics metric, shown for both ADL and FMA-UE task, with a 3.4 ± 1.1 and stride profile metric, shown for ADL walking task and *TuG* task with an average of, 3.2 ± 1.7.

**Table 3:** **Likert score results obtained during interviews (INT) in Switzerland (S) and The Netherlands (NL)**.

Interview	Stride profile	Walking speed	Reaching distance	Shoulder ROM	Reaching area	Hand distr. plot
INT S1	4	2	3	1	3	5
INT S2	3	4	3	3	3	5
INT S3	1	3	2	4	5	5
INT S4	2	4	3	0	3	4
INT S5	5	2	0	2	4	2
INT S6	5	3	2	4	3	5
INT S7	5	4	3	5	3	5
INT NL1	5	3	3	4	5	5
INT NL2	1	3	1	1	1	1
INT NL3	3	1	2	2	3	4
INT NL4	1	3	2	2	4	5
Average	3.2	2.9	2.2	2.5	3.4	4.2
SD	1.7	0.9	1	1.6	1.1	1.2

The open questionnaire was given at the end of the interview and focuses more on the INTERACTION system in general. Eight groups mentioned that using the system would probably take too much time, especially in donning and duffing the suit and set-up time. The set-up time of the system replaces regular therapy time for the patient so it needs to be beneficially for therapy. If the data capture and analysis processes can be fully automated and generate reports instantly it would be a big improvement. One group mentioned that within their private clinic, there are no resources (e.g., time and money) available to support such a system. According to three groups, originating from The Netherlands, roughly 20% of available time, in a hospital environment, can be spent on new technology or therapy (including e.g., filling in reports and data processing). In total, nine groups mentioned that it will be difficult to organize weekly get together with clinicians to discuss the CRs. They also mentioned that, in order to save time, specialists should be involved that perform the patient measurements, process the resulting data, and finally hand over reports. Three groups mentioned that in an acute hospital environment there is no advantage for such a system. The interesting part of this system is for performance measurements at home. However, in an acute care hospital environment, care professionals rarely work with patients in home situations. In ambulatory care or a rehabilitation hospital, this could be interesting technology. There are also more resources available in rehabilitation centers to do performance measurements and discuss the data with care professionals on a monthly base. One group mentioned they would rather use a strap-based system with sensors than a shirt and trousers with integrated sensing as it is easier for most patients to wear. One other group mentioned that they would like to see standardized activity of daily living tasks for training and connect patient’s deficits to these tasks. They would also like to see how many activities are performed and where those took place. Furthermore, a big data approach by incorporating different types of data (heart-rate, ECG) could be interesting.

## Discussion

A full-body inertial motion capture system, called INTERACTION, was developed by engineers and care professionals for the evaluation of the QoM of stroke patients in clinic and at home. In this usability study, we combined multiple evaluation methods, including a semi-structured interview, and questionnaires to evaluate the developed metrics among 23 care professionals from 5 different treatment centers. In this way, quantitative results on the interpretation of the metrics and the opinion of a full-body inertial motion capture system in clinic were obtained. The reference analysis results showed that most groups were able to understand, interpret, and explain the QoM metrics for two CRs by obtaining close to maximum scores. The upper extremities metrics consist mostly of new metrics, whereas for the lower extremities, presented metrics, or similar, were already known by care professionals and applied in therapy (for instance: foot clearance, which resembles heel-height stride profiles). Two metrics: reaching distance and shoulder range of motion were more difficult to apply than others related to functional movements of patients. The QoM shown within these metrics could be realized by patients in multiple ways; therefore, it remained difficult to assess the underlying cause of the QoM by the participants (35%). Hence, a combination of metrics is needed, in which the care professional can combine multiple results to come to a conclusion on the QoM of the patient.

Both the Likert questionnaire and the results from the semi-structured interviews revealed that the density plots of the hand, when applied during daily-life activities, were favored by most groups. Density plots have proven to be successful to assess upper-limb activity outside of the clinic in an earlier study by Bailey et al., 2015, stating that upper-limb activity in a patient’s real-world environment must be assessed to improve daily function (Bailey et al., 2015).

Hand distribution plots visualize not only the area wherein the patient moves their hand, but also the amount of time the hand is in a certain position. Differences between left and right can be seen instantly and in an intuitive way. Compared to current clinical methods, it gave 6 out of 11 groups more insight into the QoM of the patient and objective results which can easily be documented for the assessment of these patients over time, either numerical on in a graph/picture compared to current clinical assessments. It opens more possibilities to show progress of patients, which might not be reflected in clinical scores or the feeling of progress by the patient. According to the participants, it could give the opportunity to present visual feedback to the patient, which is important especially at home, as patients tend to lose track of progress.

The largest drawbacks in using such a full-body monitoring system are the set-up time, processing and report generation time, and the context in which data are captured at home. The first two are merely technological drawbacks. New full-body motion capture systems are currently realized, with faster set-up times and utilizing straps for attaching sensors (Xsens, 2015; Noitom, 2016). The processing and report generating algorithms developed in INTERACTION (van Meulen et al., 2016) can potentially be automated for clinical use and need to be more explored in the future. The last drawback is more challenging and involves the patient’s state (e.g., feels tired), intentions (e.g., does the patient wants to grab a piece of paper or a heavy cooking pan, or dual tasks), or surroundings (e.g., is he walking indoors or outdoors, on what type of surface, etc.). For example, participants mentioned that dual tasks clearly influence walking speed. Therefore, the patient needs to wear an additional sensor, e.g., gloves which can detect grasping an object. These were also developed in INTERACTION (Bianchi et al., 2014), but were found to be invasive to use at home as it makes daily-life activities like washing hands or go to the bathroom more difficult. Furthermore, the consistency of the surface people walk on (e.g., grass, slippery floor or sand) makes a difference for many of the lower extremity metrics like walking speed and stride profiles. These surface consistencies are difficult to detect, although promising research has been done (MacLellan and Patla, 2006; Bang et al., 2014). Within the INTERACTION project, an activity monitor was developed acting like a filter on the acquired data. It consists of basic activities like walking, sitting, standing, and arm movement (van Meulen et al., 2016). More complex activities like “brushing your teeth” or “vacuum the floor” would be a nice addition to that filter for a detailed report of activities. More so, QoM metric for specific activities can then be inspected by a care professional, assisting into a more personalized training approach for the patient.

Finally, the results suggest that a full-body inertial measurement system is most likely to be used in rehabilitation centers, rather than in (acute) hospitals as patients in rehabilitation centers are like to benefit more from such an extensive system and it remains scientifically more interesting to monitor these patients. Ambulant monitoring is not a common practice in an acute hospital division focusing on stroke survivors. This mixed method usability evaluation study has only been carried out in two countries with five different centers (hospital, rehabilitation) and included 23 care professionals. A larger analysis in more countries would be beneficial, which might reveal new insights into how stroke therapy is applied in practice, as this might differ from country to country. Certain countries could have therapeutic strategies focusing on functionality of the patient (e.g., can you pick up the cup of tea), where the others try to maximize the full capacity of the patient (e.g., can you pick it up with your arm fully stretched and body upwards).

Altogether, it remains a question how to determine what the patient is doing and where the patient is doing his or her actions. As suggested above, more body sensors can be added, but that makes a system more invasive toward the patient hence a reduced sensor system is more in favor (Kollen et al., 2006). Another way is to instrument the environment [e.g., smart home concepts and instrument objects (Hermsdorfer et al., 2013; Liu et al., 2016)]. Those systems have more issues related to privacy. It seems that in order to address the drawbacks of the current system mentioned by the care professionals for patient evaluation, a combination of body sensors and an instrumented environment is needed. Only then, it might be successfully implemented in a variety of clinics.

For future research, the current system can be reduced to a limited number of body sensors and might be combined with vibrotactile actuators for giving feedback (Klaassen et al., 2015b) and with environmental sensing which makes it possible to determine the impact of the environment on the actual QoM shown in the INTERACTION metrics.

## Author Contributions

BK performed the interviews, collected the data, and drafted the manuscript. JH, JR, and B-JB helped drafting the manuscript. HH and PV supervised the research. All the authors read and approved the final manuscript.

## Conflict of Interest Statement

The authors declare that the research was conducted in the absence of any commercial or financial relationships that could be construed as a potential conflict of interest.

## Appendix 1: Case Report Example

### Patient Example Information

Participant one is a 70-year-old right-handed woman, who lives with her husband in a flat on the third floor. Before her stroke she was working in administration and case management of a hospital. She suffered an ischemic stroke in October 2011, 12 months before participating in the study. The stroke left her with a left-sided hemiparesis. No cognitive or language impairments were detected. She is partly living independent, as she needs assistance during shopping and heavier tasks.

### Clinical Assessment Score

**Table A1:** **Clinical assessment scores for the upper and lower extremity**.

Time point	Enrollment	Discharge	Discharge + 4 weeks	Enrollment + 3 months
Fugl–Meyer total	56	63	57	58
Fugl–Meyer proximal	31	37	30	32
Fugl–Meyer distal	28	27	29	27
Action research arm test	57	56	57	57
Timed-Up-and-Go (s)	12	11	8	8
10 m walk test (m/s)	0.7	0.8	1	1.2
